# Sr and Mg Doped Bi-Phasic Calcium Phosphate Macroporous Bone Graft Substitutes Fabricated by Robocasting: A Structural and Cytocompatibility Assessment

**DOI:** 10.3390/jfb13030123

**Published:** 2022-08-23

**Authors:** Cristina Besleaga, Bo Nan, Adrian-Claudiu Popa, Liliana Marinela Balescu, Liviu Nedelcu, Ana Sofia Neto, Iuliana Pasuk, Lucia Leonat, Gianina Popescu-Pelin, José M. F. Ferreira, George E. Stan

**Affiliations:** 1 National Institute of Materials Physics, RO-077125 Magurele, Romania; 2 Department of Materials and Ceramics Engineering, CICECO, University of Aveiro, 3810-193 Aveiro, Portugal; 3 National Institute for Lasers, Plasma and Radiation Physics, RO-077125 Magurele, Romania

**Keywords:** biphasic calcium phosphate, cation substitution, robocasting, bioceramic scaffolds, bone graft substitutes

## Abstract

Bi-phasic calcium phosphates (BCPs) are considered prominent candidate materials for the fabrication of bone graft substitutes. Currently, supplemental cation-doping is suggested as a powerful path to boost biofunctionality, however, there is still a lack of knowledge on the structural role of such substituents in BCPs, which in turn, could influence the intensity and extent of the biological effects. In this work, pure and Mg- and Sr-doped BCP scaffolds were fabricated by robocasting from hydrothermally synthesized powders, and then preliminarily tested *in vitro* and thoroughly investigated physically and chemically. Collectively, the osteoblast cell culture assays indicated that all types of BCP scaffolds (pure, Sr- or Sr–Mg-doped) delivered *in vitro* performances similar to the biological control, with emphasis on the Sr–Mg-doped ones. An important result was that double Mg–Sr doping obtained the ceramic with the highest *β*-tricalcium phosphate (*β*-TCP)/hydroxyapatite mass concentration ratio of ~1.8. Remarkably, Mg and Sr were found to be predominantly incorporated in the *β*-TCP lattice. These findings could be important for the future development of BCP-based bone graft substitutes since the higher dissolution rate of *β*-TCP enables an easier release of the therapeutic ions. This may pave the road toward medical devices with more predictable *in vivo* performance.

## 1. Introduction

Synthetic bone substitutes are currently regarded as possible alternate solutions, capable of bypassing a series of important adverse effects of biological bone grafts, such as (i) morbidity derived from bone harvesting, additional pain, more complex surgical procedures, limited bone material, and onsite infections [1,2,3,4,5,6,7,8], (ii) higher risk of infection and lower structural strength [1,2,3,4,5,9], and (iii) high immunogenicity risks in the case of autografts, allografts, and xenografts [4,10], respectively. Ceramic scaffolding systems hold certain promise if several requirements are accomplished, i.e., biocompatibility, osteoconductivity, *in situ* chemical stability, lack of antigenic, teratogenic, or carcinogenic reactions, mechanical strength, and cost effectiveness [1,3,4,5,11,12]. Moreover, the scaffolds need to be porous, similar to natural bones, and accommodate cell colonization, nutrient transport, and future vascularization [1,2,12,13]. Consequently, important research efforts are currently devoted to finding suitable technologies to produce such constructs, with suitable morphostructural, compositional, mechanical, and biological properties. Methods of 3D printing (or additive manufacturing) arose as viable options since they could tackle a wide array of materials, and allow precise control over shape, morphology, and porosity. Markedly, robocasting (a technology subsidiary of the Direct Ink Writing family) rather recently emerged as a proficient technique to produce ceramic scaffolds with desired geometry and tuneable properties [14,15,16,17,18,19].

Porous calcium phosphate (CaP)-based ceramics meet most requirements of synthetic bone scaffolds, generally due to their structural and chemical similarity to the bone mineral phase [20,21,22]. Distinctly, the biphasic calcium phosphates (BCPs), i.e., blends of various proportions (in the range of 15–85 wt.%) of hydroxyapatite (HA) and *β*-tricalcium phosphate (*β*-TCP) [23,24], are considered the best materials for the fabrication of synthetic bone graft substitutes, with their *in vivo* performance depending on a specific HA/*β*-TCP ratio [14,17,25,26,27]. BCPs could represent a compromise between mechanical and biological requirements, since HA, a natural component of bone, is typically employed as the main matrix, offering mechanical strength, chemical stability in body fluids, macroporosity, and pore interconnectivity, while *β*-TCP has higher biodegradability enabling a faster release of constituent ions and thus, creating a more favourable osteogenic microenvironment [2,5,13,28]. Consequently, BCPs offer a great option for macroporous bone graft substitutes in terms of biocompatibility, osteoconductivity, and controlled resorption rate, while maintaining pore dimension and interconnectivity at a bone-like level, so that the nutrients and metabolites can circulate easily, enabling bone-tissue regeneration [22,29].

Furthermore, increasing evidence indicates that the biofunctionality (e.g., osteoconduction, angiogenesis, rapid healing, and antibacterial effects) of CaP-based ceramics can be augmented and even be extended by designed cation and anion substitutions [17,30,31,32]. In fact, the major inorganic component of natural bone is also a multidoped calcium phosphate, containing both cations and anion substituents (i.e., Na^+^, F^−^, Cl^−^, K^+^, Mg^2+^, Fe^2+^, Zn^2+^, Sr^2+^, Cr^3+^, Si^4+^) [33], and so, the process of BCP doping could be seen as a nature-inspired solution aimed to boost its biofunctions. Importantly, ionic substitutions are also considered noteworthy alternate solutions to the use of recombinant growth factors for inducing osteogenesis and angiogenesis [34,35,36]. Although promising results were obtained, concerns regarding the safety of recombinant growth factors emerged and thus, their full-scale implementation is still hindered [37,38]. This, together with the complexity of their individual responses, high-cost, and intricate usage protocols, endorses the simpler, safer, and economically advantageous (higher volume and lower costs) path from research to bedside of therapeutic ion substitutions [34,35].

For this work, magnesium (Mg) and strontium (Sr) were selected as substituents into BCP ceramics, due to their advertised biological benefits. Collectively, there is a current consensus that Mg doping of CaPs can stimulate bone-like apatite growth in simulated body fluids media and conserve or improve cell adhesion and proliferation while eliciting antibacterial effects markedly against *Staphylococcus aureus* (ATCC 29213), *Escherichia coli* (ATCC 25922), and *Pseudomonas aeruginosa* (ATCC 27853) bacterial strains [30,31,39]. Furthermore, *in vivo* tests showed that magnesium (Mg)-doping of *β*-TCP- and BCP-based scaffolds promotes new bone formation and angiogenesis [22,40,41]. On the other hand, Sr is known to improve biomineralization capacity, stimulate osteogenesis and osseointegration, enhance bone regeneration, and decrease bone resorption [41,42,43,44,45,46,47,48], while inducing in some cases antibacterial effects [30,31,41,42,43,44,49,50] against *Actinomyces viscosus* (ATCC 19246), *S. aureus* (ATCC 28923, ATCC 6538) and *E. coli* (ATCC 8739) [30,45,46,47]. Most importantly, recent evidence suggests that multielement doping could play a synergistic biofunctional role, and thus, the simultaneous cation substitution is currently envisaged as a next-generation powerful tool for bone regeneration [31,50,51,52].

Hence, this study was devoted to a comparative multiparametric—morphostructural and preliminarily *in vitro* biological—analysis of undoped, single- (Sr) and double- (Sr and Mg) doped BCP macroporous scaffolds for bone repair and regeneration in orthopaedic, dentistry, maxillofacial surgeries, and tissue engineering applications. Four main goals were specifically targeted: (i) selection of the compositions and the synthesis of the starting BCP powders (undoped, single-doped, and co-doped (Sr, Mg)); (ii) design and robocasting fabrication of tailor-made BCP-based porous scaffolds for an efficient cell seeding; (iii) reveal the incorporation preferences and structural roles of Mg and Sr in the BCP phase components (which is sporadically tackled in literature and might prove important for the future understanding and predictability of their biological performance); and (iv) perform the first cytocompatibility screening tests of these single (Sr) and double (Mg and Sr) doped bioceramic scaffolds as a selection tool for future complex biological assessments, which could pave the road toward improved bone graft substitute designs.

## 2. Materials and Methods

### 2.1. Synthesis of Biphasic Calcium Phosphate Powders

Simple, Sr-substituted (Sr/(Sr + Ca) = 6 at.%) and Sr (Sr/(Ca + Sr + Mg) = 6 at.%) and Mg (Mg/(Ca + Sr + Mg) = 2 at.%) doubly-substituted bi-phasic (HA + *β*-TCP) calcium phosphate powders were synthesized using the hydrothermal method, following a protocol developed in previous studies [17,53]. The sample codes along with their anticipated composition are presented in Table 1.

The concentration of doping elements was decided based on the vast experience of the research team in the specific field of ionic substitutions in CaPs (e.g., [54,55,56,57]) and on two relevant material design prerequisites: 

(i) Mg is a minor element of the bone mineral component which generally has a concentration (Mg/(Mg + Ca)) of <2 at.% [58,59] and plays a prominent role in bone metabolism. Its deficiency could determine a series of possible adverse effects such as the inhibition of bone growth, loss of bone mineral density, and increase of bone fragility [30,60].

(ii) Although Sr is just a trace element in bone [58,59], it has a higher half-maximal cytotoxic concentration [31], and is also widely applied as medication (i.e., *Strontium ranelate*) for the treatment of osteoporosis in elderly patients, increasing bone formation, and decreasing bone resorption [61,62].

High purity (≥99.0%) reagents were selected for the synthesis of the powders. Briefly, Ca(NO_3_) · 4H_2_O and (NH_4_)HPO_4_ (AppliChem, Darmstadt, Germany) were used as the calcium and phosphorous precursors. In the case of the single Sr- and Sr–Mg-double substituted CaPs, Sr(NO_3_)_2_ and Mg(NO_3_) · 6H_2_O (Sigma-Aldrich, St. Louis, MO, USA) were employed. CO(NH_2_)_2_—urea—(Riedel-de Haën, Honeywell Research Chemicals, Seelze, Germany) was selected as a pH controller agent. 

The cation (i.e., calcium, calcium and strontium, or calcium and strontium and magnesium) and anion (i.e., phosphorous) precursor solutions were prepared independently, using deionized water as a solvent. To the latter, 0.5 M of CO(NH_2_)_2_ was also added. These two separate solutions were further mixed at a cation–phosphorous atomic ratio of 1.57 and put under continuous magnetic stirring in an autoclave at a temperature of 150 °C for 4 h, and then allowed to cool down to room temperature overnight. Subsequently, the as-prepared suspensions were filtered, and the resulting wet cakes were dried at 100 °C for 24 h in a forced air oven. The resulting powders were calcined for 2 h at 1100 °C, using a heating rate of 5 °C/min, followed by natural cooling inside the furnace, and then grounded in a high-energy agate ball mill for 35 min. The particle size distributions (PSDs) curves of the heat-treated and ball-milled powders have been already reported [53]. Their median (D_50_) particle sizes were within the narrow range of ~1.2–1.4 μm [53]. After being deconvoluted, PSDs were revealed to consist of three main populations, the finest one centred at ~0.55 μm; an intermediate and most predominant population centred at ~2.4 μm; and a coarser fraction of partially destroyed particle agglomerates with sizes ≤8 μm [53].

### 2.2. Preparation of the Robocasting “Inks” and Printing of the 3D Macroporous Constructs

Prior to the preparation of inks and pastes, all three ball-milled BCP powders were sieved through a 63 μm-sized mesh for retaining eventual coarser agglomerates. The inks consisted of aqueous suspensions loaded with the BCP-based powders prepared according to the protocol described in sub-Section 2.1. A traditional approach was applied, by adding successively designed (minimized) amounts of aqueous solutions (aq. Sol.) of anionic polyelectrolyte dispersant (Targon 1128—an ammonium polyacrylate salt, BK Giulini GmbH, Ladenburg, Germany), binder–viscosifying agent (hydroxypropyl methylcellulose—HPMC, number average molecular weight M_n_ ≈ 10,000, Sigma-Aldrich, St. Louis, MO, USA), and cationic flocculant-coagulating agent (polyethyleneimine—PEI, number average molecular weight M_n_ ≈ 1800, Sigma-Aldrich, St. Louis, MO, USA), transforming step-wise the mixtures into pastes with pseudoplastic comportment (shear-thinning under stress). The homogenization process was carried out in a dedicated Thinky ARE-250-CE planetary mixer (Thinky USA Inc., Laguna Hills, CA, USA). The refined paste recipes are presented in Table 2.

The BCP-based scaffolds were printed using a 3D Inks (model EBRD-A32) robocasting mechanical motor-driven system (3D Inks, LLC, Tulsa, OK, USA) equipped with 3 mL Nordson EFD syringes (Nordson Corporation, Westlake, OH, USA) with Luer-type nozzle lockers. All BCP-based pastes were extruded through Nordson EFD 250 μm-diameter nozzles (Nordson Corporation, Westlake, OH, USA) at a speed of 10 mm/s. Cylindrical scaffolds, with a diameter of 8 mm and a height of ~6 mm, were fabricated by depositing 24 consecutive layers using repeated printing patterns in a 45 degrees-rotation design. The distance between two successive extruded filaments was 300 μm. This scaffold design was selected to obtain a complex tortuosity of the macroporous construct, which could hypothetically enable an improved cell colonization and mimic more accurately the intricate internal structure of bone trabeculae. These tailor-made constructs were empirically designed [17] to have an interconnected macroporosity of ~45–50% situated in the realm of the trabecular total porosity which spans in the range of 30–90% [63].

The green scaffolds were allowed to dry at room temperature for 24 h and then subjected to calcination at 600 °C/1 h (to eliminate the organic additives) and sintering at 1100 °C/4 h, using heating rates of 1 and 5 °C/min, respectively. After the end of the calcination–sintering program, the furnace was turned off, and the scaffolds cooled down to room temperature, naturally, in ca. 12 h. Parts of the scaffolds were crushed and grounded into fine powders using an agate mortar and pestle for further compositional and structural analysis.

### 2.3. Physical–Chemical Characterization Techniques

The particle size and particle-size distribution of the source powders were inferred by dynamic light scattering (DLS) measurements using the Beckman Coulter LS230 apparatus, (Beckman Coulter Inc., Brea, CA, USA) and applying the Fraunhofer optical model.

The morphology of robocasting scaffolds was analysed using field emission scanning electron microscopy (FE-SEM) with the help of a Carl Zeiss Gemini 500 system (Carl Zeiss Company, Oberkochen, Germany), on both the cross-section (using fractured samples) and the surface of the constituting filaments. No conductive coating was applied. The FE-SEM images were collected under a high vacuum (~10^−^^4^ Pa) at a working distance of 11 mm and an acceleration voltage of 2 kV.

The chemical composition of the BCP-based 3D-printed constructs was determined by energy dispersion X-ray spectroscopy (EDXS) using an EDAX Inc. (Mahwah, NJ, USA) system, operated at an acceleration voltage of 10 kV. The EDXS investigations were performed on four, randomly chosen, large regions (with areas of 250 × 250 μm^2^) of each specimen. A hydroxyapatite NIST (National Institute of Standards and Technology) SRM (Standard Reference Material) no. 2910b was used as a reference sample.

The structural vibrational features and presence of functional groups were surveyed using Fourier-transform infrared (FTIR) spectroscopy in attenuated total reflectance (ATR) mode, using a Jasco 6800-FV-BB spectrometer (Jasco Corporation, Tokyo, Japan) with an ATR PRO670H system with diamond crystal. Both the BCP-based (grounded) scaffolds and control samples (i.e., hydroxyapatite NIST-SRM 2910b and commercial pure *β*-TCP Sigma-Aldrich, product number 49963) were analysed. The FTIR-ATR spectra were recorded under vacuum in the spectral range of 100–2000 cm^−1^, at a resolution of 4 cm^−1^. The presented spectra represent the average of 64 individually collected scans. An ATR correction (anomalous dispersion mode) was applied to the spectra.

The crystalline quality of samples was analysed using X-ray diffraction in Bragg–Brentano geometry using a Bruker D8 Advance diffractometer (Bruker AXS Advanced X-ray Solutions GmbH, Karlsruhe, Germany), with nickel filtered CuK*_α_* radiation (*λ* = 1.5418 Å) and a Bruker LynxEye^®^ 1D detector operated in integration mode. The patterns were acquired in the 2θ range 9–70°, with a step of 0.02°, and an acquisition time of 1 s/step. The instrument was calibrated with a corundum NIST-SRM 1976 standard reference sample. The determination of lattice parameters, average crystallize size, cation site occupancy, and constituting phase weight were determined by Rietveld refinement using the MAUD diffraction data processing program (v2.992).

### 2.4. In Vitro Cytocompatibility Assays

The cytocompatibility assessment of the BCP-based scaffolds was tested in hFOB 1.19 human osteoblasts (ATCC^®^ CRL-11372) cell cultures, due to the targeted potential use of these macroporous scaffolds as bone graft substitutes in the reconstruction of 3D osseous defects. The hFOB 1.19 cells were considered the closest *in vitro* model for the final intended purpose.

#### 2.4.1. Sample Preparation

The scaffolds were sterilized in glass vials with dry heat (1 h at 180 °C) and transferred in sterile Millicell 24-well plates with polycarbonate cell culture inserts with a pore size of 0.4 μm (Merck Millipore, Burlington MA, USA)—optimized to grow and sustain high integrity cell monolayers—in the cell culture laminar flow hood.

#### 2.4.2. Human Osteoblasts Cell Culturing

The hFOB 1.19 human osteoblasts (ATCC^®^ CRL-11372) cells were maintained in culture according to provider indications (LGC Standards Ltd., Teddington, UK), using phenol-free Dulbecco’s Modified Eagle’s Medium Nutrient Mixture F-12 Ham 1:1 (DMEM/F12, Sigma-Aldrich, St. Louis, MO, USA) supplemented with 10% foetal bovine serum and G418 (Geneticin) 1×, at a low growing temperature of 34 °C, in order to maintain an undifferentiated state of cells. Cells were detached using trypsin, counted with a Reichert hemacytometer, centrifuged for 10 min at 225× *g* and resuspended in complete culture media at a density of 2 × 10^6^ cells/mL. A volume of 25 μL of medium containing 5 × 10^4^ cells was layered atop of the scaffolds and allowed to penetrate the capillaries formed inside the macroporous structure. Upon seeding, the scaffolds were turned to one side in order to facilitate uniform seeding inside the constructs and to counteract the falling of the cells through the vertical pores at the bottom of the scaffold in contact with the polycarbonate surface of the wells. The scaffolds were transferred in the cell culture incubator to facilitate adherence of the cells to the inner structure of the scaffolds. After 4 h, 1 mL of fresh cell culture media was added to each well, with the level of medium surpassing the top surface of the scaffolds. After another 24 h, the cell culture medium was removed, and 1 mL of fresh cell culture medium was added. The cells were maintained in culture for 14 days. A single 7-day cell-medium change was preferred to ensure that the cells would be subjected to high ion concentrations leached from the BCP scaffolds for the longest time possible (at the same time preventing the depletion of nutrients in the medium which were necessary for cell development). 

In each experiment or assay (presented hereunder), the control was constituted by an equivalent number of cells seeded in wells with no scaffolds of the same cell culture plate (with a highly adherent cell culture surface) with a complete medium and no supplemental factors.

#### 2.4.3. MTS-Based Cell Proliferation Assay

Fourteen days after cell seeding, the growth medium was discarded from each well and changed with an assay buffer volume of 960 μL prepared according to the producer’s (Promega Corporation, Madison, WI, USA) recommendations (i.e., 800 μL fresh phenol red-free medium and 160 μL of (3-(4,5-dimethyl thiazol-2-yl)5-(3-carboxymethoxyphenyl)-2-(4-sulfophenyl)-2H-tetrazolium)—MTS ready-to-use buffer). The level of the medium was above the sample’s height. The plates with the samples were transferred in the cell culture incubator on an orbital shaker at one revolution. The orbital shaker was needed in order to ensure the homogenization of all the mediums inside and outside the scaffold. In the absence of shaking, gradients were created and errors could appear. The cells consumed the MTS substrate leading to its relative scarcity deep into the scaffold capillaries, in parallel with the accumulation of the formazan compound that can ultimately lead to different reaction equilibriums in the scaffold samples and the polycarbonate control wells. Additionally, a greater concentration of formazan may cause its absorption on the material surface. After 1 h incubation, volumes of 120 μL were transferred in a 96-well plate. The absorption at 490 nm was read with a Zenith 3000 (Anthos Labtec Instruments, Salzburg, Austria) multimode reader. The MTS-based cell proliferation assays were performed in sextuplicate. 

#### 2.4.4. Lactate Dehydrogenase (LDH)-Based Cell Proliferation Assay

After 14 days of hFOBs culturing on BCP-based scaffolds and polycarbonate, 100 μL of lysis buffer was added to each well. Further, the wells of the plate were covered with a plate sealing film (EASYseal™, Sigma-Aldrich, St. Louis, MO, USA), the lid placed and secured with Parafilm^®^ M (Carl Roth GmbH + Co, Karlsruhe, Germany) and transferred in an ultrasonic bath (Fisherbrand, CPXH series, model 1800, Fisher Scientific, Waltham, MA, USA) with water containing ice flakes, operated at 40 kHz and 150 W for 30 min. This way, lysis of all cells was obtained, even in conglomerates deep in the scaffold. The sonication also allowed for a uniform distribution of LDH enzymes in the medium. A volume of 50 μL of medium was transferred in 96-well plates and subjected to LDH analysis according to the kit manufacturer (Promega Corporation, Madison, WI, USA) protocol. The absorptions were measured at 450 and 620 nm with the Zenith 3000 (Anthos Labtec Instruments, Salzburg, Austria) multimode reader. For each situation, the result was obtained by observing the difference between the 450 nm absorption and the reference absorption obtained at 620 nm. All data are presented as a percent from the polycarbonate control. The LDH-based cell proliferation assays were performed in sextuplicate. 

#### 2.4.5. Acridine Orange (AO)-Based Cell Proliferation Assay

After 14 days of hFOBs culturing, the culture medium was changed with fresh 1 mL of culture medium containing 0.4 μM of AO. The plates were introduced in the cell culture incubator for 15 min to allow the penetration of the fluorescent tracer in the cells. The medium containing AO was discarded, and the samples were washed three times with fresh medium. The scaffolds were transferred in capped test tubes containing 1.5 mL detergent-based lysis buffer (containing 1× concentration of ProtectRNA™ RNases inhibitor 500× concentrate, Sigma-Aldrich, St. Louis, MO, USA) and maintained for 30 min in an ice-cold ultrasonic bath (Fisherbrand, CPXH series, model 1800, Fisher Scientific, Waltham, MA, USA), operated at 40 kHz and 150 W, in order to extract and homogenize the AO coupled with RNA. A volume of 120 μL was transferred in a 96-well fluorescence plate (with black walls and a glass bottom). The plates were read with the Zenith 3000 (Anthos Labtec Instruments, Salzburg, Austria) multimode reader, using a single well protocol average of 10 bottom reads, 450 nm excitation filter with a 20 nm bandpass, 530 nm emission filter with a 30 nm bandpass. Results are presented as a percent of the control cells cultivated in polycarbonate wells that were considered as 100%. The AO-based cell proliferation assays were performed in sextuplicate. 

#### 2.4.6. LDH Cell Death Assay

After 14 days of culture, 50 μL of cell culture medium was harvested from each well and transferred to 96-well plates. A volume of 50 μL of assay buffer, freshly prepared according to the producer (Promega Corporation, Madison, WI, USA) indications was added to each well and the plate was transferred in the cell culture incubator. After 30 min, 50 μL of stop buffer was added to each well and plates were read in with the Zenith 3000 (Anthos Labtec Instruments, Salzburg, Austria) multimode reader. The results were inferred from the difference between the absorption read at 450 nm and the control absorption read at 620 nm. The percent of dead cells was obtained by calculating the percentage of the LDH quantity found in the cell growing medium versus the LDH quantity found in all the cells that have grown in a specific well for the 14 days of the experiment. The LDH cell death assays were performed in triplicate. 

#### 2.4.7. Epifluorescence Microscopy

After 14 days of cell culturing, the medium was removed and 1 mL of ice-cold fixation reagent (4% formalin in phosphate-buffered saline (PBS)) was added. After 15 min, three 10 min washes with PBS were carried out. A volume of 1 mL of 1× solution of Phalloidin AlexaFluor 546 (Invitrogen, Carlsbad, CA, USA) and 0.5 μg/mL of 4′,6-diamidino-2-phenylindole (DAPI) (Sigma Aldrich, St. Louis, MO, USA) in PBS was added to each well. After 1 h of incubation with the fluorescent tracer, two PBS washes of 10 min each were performed. The scaffolds were analysed after a 15 min final wash with double distilled water. The morphology of the cells grown in the scaffolds was investigated under a Leica DM6 B epifluorescence microscope (Wetzlar, Germany), equipped with a Leica DFC 9000 GT camera, the appropriate fluorescence objectives, and filters, and the LASX software suite.

#### 2.4.8. SEM Preparation and Examination

After 14 days of culturing, the medium was removed and the cells were fixed for 1 h with 1 mL of ice-cold freshly prepared 4% glutaraldehyde solution. After a set of two 10 min washes with PBS, a dehydration protocol was applied by submerging the scaffolds in vials with rising ethanol in water concentrations of 70, 95, and 100%. After the pure ethanol treatment, the scaffolds were submerged in xylene for 10 min and allowed to dry in a chemical hood. The morphology of the cells grown in the scaffolds was analysed with FE-SEM using a Carl Zeiss Gemini 500 (Carl Zeiss Company, Oberkochen, Germany) microscope. No conductive coating was applied. The FE-SEM images were collected at a working distance of 5 mm and an acceleration voltage of 1 kV. 

#### 2.4.9. Statistical Analysis

The statistical analysis for the cell proliferation and cell death responses was performed using the one-way ANOVA multiple analysis comparison followed by a Tukey’s post hoc test with the help of the GraphPad Prism v.9.4.1 software (GraphPad Software, San Diego, CA, USA). Differences were considered statistically significant at a *p*-value < 0.05. In parallel, a two-tailed distribution unequal variance Student’s *t*-test, with the same fixed *p*-value cut-off of 0.05 for significance, was carried out for comparison, employing the same statistical analysis software. With one exception (which is highlighted in sub-Section 3.2.2.), the two analysis procedures provided the same statistically significant differences between the studied situations.

## 3. Results and Discussion

### 3.1. Physical-Chemical Investigations

#### 3.1.1. Morphology and Composition

**Figure 1:**
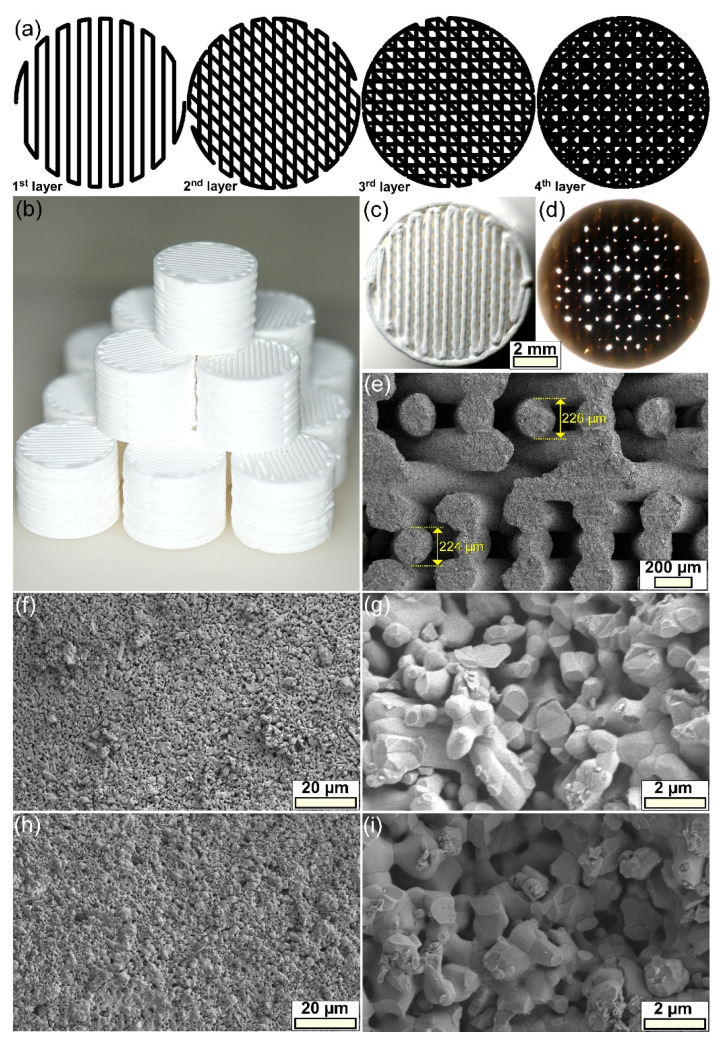
(**a**) The schematic portrayal of the printing pattern employed for the fabrication of the scaffolds, using a 45 degrees-rotation design. (**b**) Representative batch of as-fabricated scaffolds. (**c**,**d**) Optical photographs of a scaffold illuminated from (**c**) above and (**d**) below, demonstrating the projected macroporosity pattern. (**e**) General SEM image of a scaffold fractured along its height. (**f**–**i**) FE-SEM microstructure of the (**f**,**g**) BCP- and (**h**,**i**) BCP-6Sr2Mg-based scaffolds, evidenced on the (**f**,**h**) surface and (**g**,**i**) cross-section of the ceramic filaments.

At a macroscopic level, it was demonstrated that the intended scaffold architecture and its macroporosity design (Figure 1a,b) was reproduced well by overlapping, in a controlled pattern via robocasting technique, the ceramic filaments (Figure 1c,d). This represented the first indication of the ability of the printed filaments to retain their shape and support the weight of layers deposited in subsequent fabrication steps. This observation was further strengthened by microscopic level investigations, carried out by SEM at low magnification (Figure 1d) on fractured scaffolds. Both the good spherical conformity of the ceramic filaments and the reduced volume of contact (interpenetration) between intersecting filaments, was revealed. Furthermore, the diameter of the filaments was situated in a narrow dimensional range, with an average of ~225 μm, unveiling a sintering contraction of ~10%. The higher magnification FE-SEM analyses (Figure 1f–i) showed that the ceramic filaments composing the scaffolds consisted of polyhedral-shaped grains with sizes of ~0.4–1.2 μm. Additionally, an internal (intergranular) microporosity (~0.2–0.9 μm) was highlighted both on the surface (Figure 1f,h) and in the volume (Figure 1g,i) of the printed ceramic filaments. These microstructural features were analogous for all types of scaffolds, irrespective of their composition. The microporosity, assessed with the help of the Image J software (National Institutes of Health, Bethesda, MD, USA), using multiple SEM images, was estimated at ~9–10%, regardless of the type of scaffold.

The 3D reconstruction of the sintered scaffolds, completed in SolidWorks 3D CAD 2019 software (Dassault Systèmes, Vélizy-Villacoublay, France), considering their architecture (i.e., diameter, number of layers, total height) and geometrical information extracted from SEM images (i.e., the diameter of filaments, spacing between two consecutive filaments, interpenetration) confirmed the empirically predicted macroporosity (~45%). Further, quantitative micro- and macroporosity evaluations were performed based on density determinations using Archimedes’ principle, with water employed as the liquid medium. Density values of ~2.95–3 g/cm^3^, and thus densifications in the range of ~93–95%, were obtained irrespective of the type of scaffold, pointing to microporosity values of ~5–7%, thereby, close to the ones estimated based on SEM image analysis. The ratio of the scaffolds’ masses weighted in air and the as-determined density values allowed the inference of the volumes of the macroporous scaffolds. When subtracting these values from the volumes of solid cylinders with the external sizes of the porous scaffolds (measured with a calliper), the aggregate micro- and macroporosity was estimated. This was found to be situated in the narrow range of ~51–53%, denoting a macroporosity level of ~46–48%, thus, close to the expected one. 

While the macroporosity ensures cell penetration, colonization, migration, and adequate flow of nutrients within the scaffold [64,65], the microporosity is now known to play a central role in the biofunctional response of an implanted material, fostering noteworthy benefits [66,67,68,69]. A microporous surface, in addition to the understandable increase of the active surface area (for cells to attach to), endowed the biomaterial with highly relevant properties, by increasing the number of protein absorption sites or creating capillary forces which can boost cells’ adherence [66,67]. This way, a more intimate scaffold cell interaction was generated, fast-tracking the degradation of the biomaterial and the release of therapeutic agents within [66,67]. Although a direct interdependence between the microporosity and tissue regeneration rate seems to be implied, this specific material feature cannot be increased without the inherent decrease of the overall mechanical performance of the scaffolds [70,71,72,73]. Several attempts have been made to identify an optimum microporosity for scaffolds [67,69,74,75] with findings being rather inconsistent. This furthermore suggests that an inter-relationship between microporosity, average pore size, and biological response cannot disregard the type of constituting material and its intrinsic properties (e.g., composition, crystallinity, surface energy, and degradability). In the closest report to our study, Rosa et al. [75] produced HA-based scaffolds with a small pore size (<10 μm) and three different degrees of microporosity of 5, 15, and 30%, sintered at temperatures in the range of 1150–1250 °C. Superior cell proliferation rates were yielded by the scaffolds with microporosity degrees of 5 and 15%.

**Figure 2:**
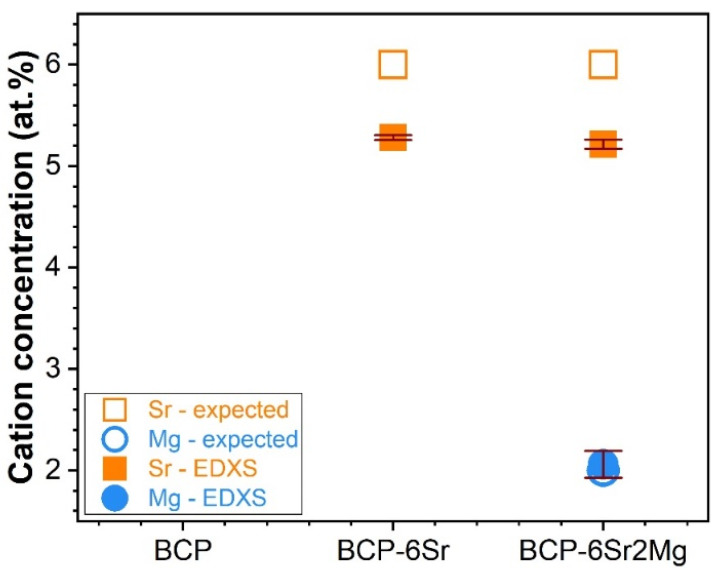
Anticipated and experimental (determined on the basis of EDXS analyses) cation (Mg^2+^ and Sr^2+^) concentrations of the CaP-based materials, expressed as Sr/(Ca + Sr)*100 at.% (for BCP-6Sr) or Sr/(Ca + Sr + Mg)·100 at.% and Mg/(Ca + Sr + Mg)·100 at.% (for BCP-6Sr2Mg).

The concentration of the Mg and Sr cation substituents was inferred by EDXS, and the results were plotted in Figure 2 against the anticipated (intended) contents. If the ionic concentration of Mg was fairly reproduced (with only a ~3% relative increase being recorded), then for both the single- and double-doped CaP materials, a significantly larger deviation of Sr (i.e., a ~13% relative decrease with respect to the anticipated concentration) was revealed. These results agree with previous studies on cation-doped CaP-based bioceramics synthesized by wet-chemical routes, which evidenced a drop in the Sr incorporated concentration at contents equal to or in excess of 5% [76]. It is thus suggested that the solubility limit (threshold) of Sr in the lattice of CaPs was reached in the vicinity of this value, with the Sr excess being washed away in the filtration step.

#### 3.1.2. Structure

**Figure 3:**
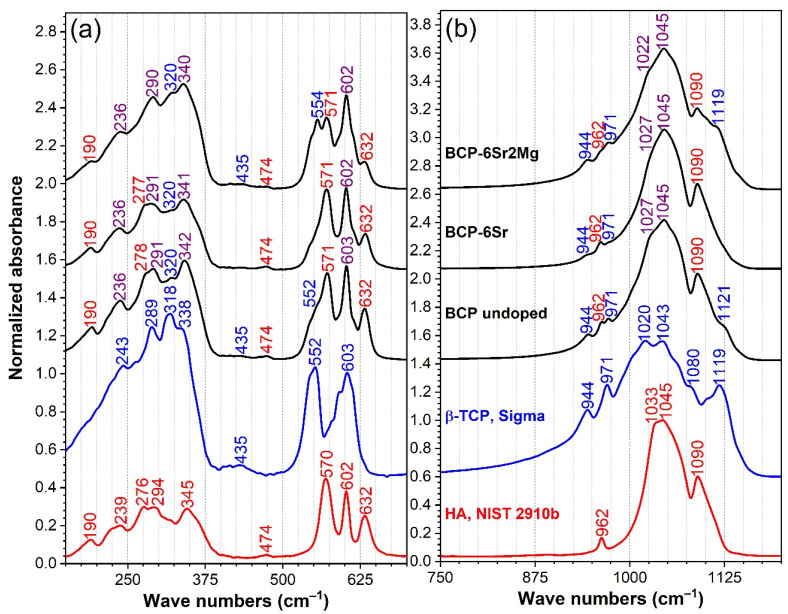
The comparative FTIR-ATR spectra of the BCP-based materials, presented in two relevant wave number regions: (**a**) 150–700 cm^−1^ and (**b**) 750–1200 cm^−1^. The spectra of two control samples, i.e., a hydroxyapatite NIST-SRM 2910b specimen (red curve) and a *β*-TCP Sigma-Aldrich (blue curve) pure commercial material (Sigma-Aldrich, product no. 49963), were inserted at the bottom of each graph frame for reader’s reference.

The FTIR-ATR spectra of the CaP specimens (obtained by grinding scaffolds from each batch into fine powders) are presented comparatively with respect to high-purity hydroxyapatite (NIST-SRM 2910) and *β*-TCP (Sigma-Aldrich) specimens in Figure 3. There are obvious differences between the FTIR spectra of HA and *β*-TCP materials, which could be used for the qualitative phase composition assessment of blended and assorted materials. 

In the mid-IR fingerprint region (stretching down to 400 cm^−1^) the HA and *β*-TCP elicited their characteristic IR absorption bands (Figure 3a,b), namely:

- HA presented an IR absorption spectrum featuring sharp peaks assigned to the doubly-degenerated (ν_2_) bending (~474 cm^−1^), triply-degenerated (ν_4_) bending (~570 and 602 cm^−1^), nondegenerated (ν_1_) symmetric stretching (~962 cm^−1^), and triply-degenerated (ν_3_) asymmetric stretching (~1033, 1045 and 1090 cm^−1^) of the orthophosphate groups; and the libration (ν_L_) of the structural hydroxyl units (~632 cm^−1^) [76,77,78].
- β-TCP yielded a much more convoluted spectral envelope presenting broad maxima associated with the doubly-degenerated (ν_2_) bending (~435 cm^−1^), triply degenerated (ν_4_) asymmetric bending (at ~552 and 603 cm^−1^), factor group splitting of (ν_1_) symmetric stretching (at ~944 and 971 cm^−1^), and the triply degenerated (ν_3_) asymmetric stretching (at ~1015, 1036, 1081, and 1116 cm^−1^) of orthophosphate units [78,79,80].

The far-IR region (<400 cm^−1^) of the HA and *β*-TCP was less explored to date, and it is known to appertain to the crystal lattice mode bands. The band peaking at ~345 cm^−1^, evident only in the case of HA, is generated by the translational and sublattice 2[(Ca_3_–OH] modes [77,81]. At lower wave numbers, both HA and *β*-TCP spectra are marked by a series of weak vibrational bands which can be associated with the lattice motions (primarily libration modes) of Ca–PO_4_ [77,78,79,80,81].

As a general observation, all FTIR-ATR spectra are characterized by a well-defined, narrow, and split aspect of the IR absorption bands, which is indicative of a good crystallization of the prepared CaPs [77]. The complex shape of the spectral envelopes of the prepared simple- and cation doped-CaP samples, suggested their blended HA—*β*-TCP composition. The maxima of the IR absorption peaks were coloured in red (for HA), blue (for *β*-TCP), and violet (for superimposed HA and *β*-TCP vibrational modes) (Figure 3). By comparing the amplitude of the distinct (not superimposed) bands safely associated with the vibration modes of either HA or *β*-TCP, it can be deduced that BCP-6Sr2Mg has undergone a major structural transformation, with the *β*-TCP phase becoming now the prominent one. Specifically, the ~544 cm^−1^ (determined by one of the triply degenerated (ν_4_) asymmetric bending modes of *β*-TCP), ~944 and 971 cm^−1^ (belonging to the factor group splitting of (ν_1_) symmetric stretching modes of *β*-TCP), and the ~1119 cm^−1^ (owned to one of the triply degenerated (ν_3_) asymmetric stretching modes of *β*-TCP) peaks increased in intensity at the expense of an evident amplitude reduction of the ~571 cm^−1^ (determined by one of triply degenerated (ν_4_) asymmetric bending modes of HA), ~962 cm^−1^ (belonging to the (ν_1_) symmetric stretching mode of HA), and ~1090 cm^−1^ (owned to one of the triply degenerated (ν_3_) asymmetric stretching modes of HA) maxima.

**Figure 4:**
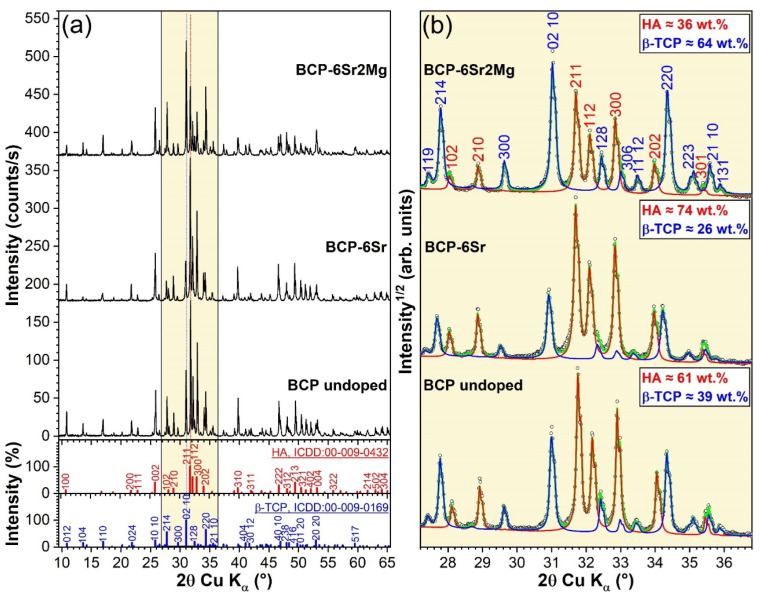
(**a**) Comparison of the XRD patterns of the BCP-based materials, presented together with the ICDD-PDF4 reference diffraction files of the HA and *β*-TCP constituents. (**b**) Zoomed angular 2θ (27.5–35.5°) region of the XRD diagrams of the highest intensity diffraction maxima of HA and *β*-TCP phases, along with their corresponding Rietveld fits. The HA and *β*-TCP phase compositions (in wt.%) of each type of BCP specimen are embedded in the graphs. Note: circles—measured data; red and blue curves—simulated patterns of HA and *β*-TCP, respectively; green curve (sent back of data)—total calculated or simulated XRD pattern.

The XRD patterns of the prepared CaP-based samples are shown comparatively in Figure 4a, with respect to the reference ICDD-PDF4 files of HA (no. 00-009-0432) and *β*-TCP (no. 00-009-0169). All samples were mixtures of HA and *β*-TCP, their diagrams yielding sharp diffraction peaks (indicative of their good crystalline quality). Thereby, in good agreement with the aforepresented FTIR-ATR spectroscopy findings (Figure 3), the BCP nature of the specimens was further confirmed by XRD. The lack of additional crystalline phases provided the first evidence that the dopants (i.e., Mg and Sr) have been fully incorporated into the HA and *β*-TCP lattices. A zoom in 2θ region (~27.5–35.5°) of the prominent HA and *β*-TCP diffraction maxima (Figure 4b), allowed one to observe, solely based on their relative qualitative intensity change, that the *β*-TCP/HA ratio increased in the order BCP-6Sr < BCP < BCP-6Sr2Mg. The quantitative phase analysis, performed by Rietveld analysis gave *β*-TCP concentrations of 64, 26, and 39 wt.% for the undoped-BCP, BCP-6Sr, and BCP-6Sr2Mg samples, respectively. Figure 4b emphasizes the excellent fit of the experimental data with the simulated patterns obtained by Rietveld refinement. The calculated errors of the weight percentages were below 1%. 

Furthermore, a noteworthy shift of the *β*-TCP peaks of BCP-6Sr towards lower angles was noticed (Figure 4b) with respect to undoped BCP. The incorporation of ~2 mol% Mg (i.e., BCP-6Sr2Mg samples) resulted in the return of the *β*-TCP peaks closer to the reference positions. The HA peaks of both the simple- and double-doped BCP samples elicited a low-angle shift similar to the one recorded in the case of the *β*-TCP counterpart of BCP-6Sr.

**Figure 5:**
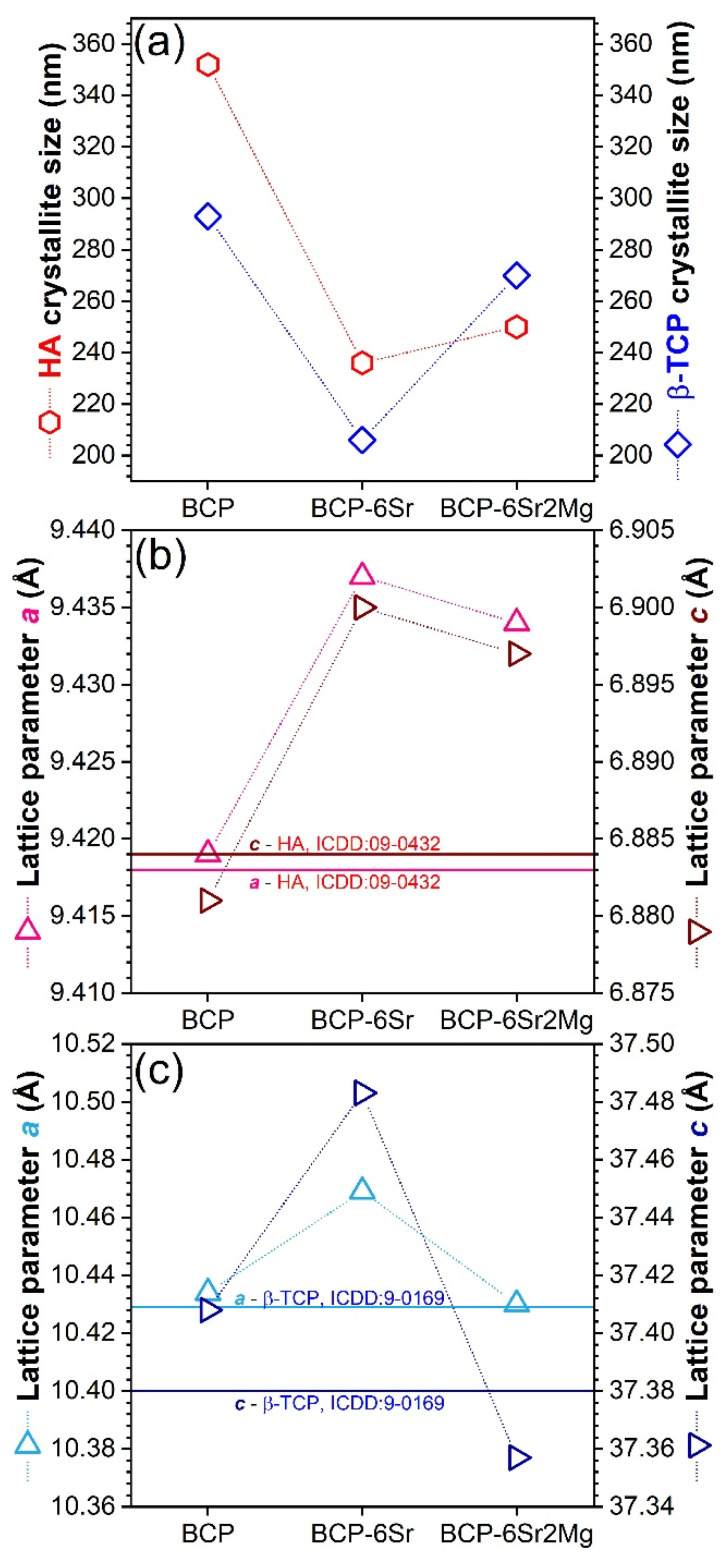
(**a**) Average crystallite size of the HA and *β*-TCP phases. (**b**,**c**) Evolution of the *a*- and *c*-axis lattice constants of the (**b**) HA and (**c**) *β*-TCP phases. Notes: The solid horizontal lines in (**b**) and (**c**) indicate the a- and c-axis lattice parameters values stipulated in the ICDD-PDF4 reference diffraction files of HA and *β*-TCP. The error bars of Rietveld-refined structural parameters were not represented, since their values were too small to be visualized well.

In the undoped BCP, the crystallite sizes (coherence lengths) of HA and *β*-TCP were ~350 nm and ~300 nm, respectively (Figure 5a). By assuming anisotropic crystallite shapes in MAUD and using the “Popa model” approach [82], it was revealed that in both the undoped and doped BCP materials, the HA crystallites were slightly flattened in the direction of the *c*-axis, while the *β*-TCP crystallites were nearly spherical. The crystallites were smaller in the case of doped BCPs. Specifically, in the case of (i) Sr- and (ii) Sr- and Mg-substituted BCPs, the HA and *β*-TCP constituents had mean crystallite sizes of (i) ~240 and ~200 nm and (ii) ~250 and ~270 nm, respectively (Figure 5a). Hence, it appears that Mg favoured the *β*-TCP crystallite growth.

The Sr single doping increased the *a*- and *c*-lattice constants of both HA and *β*-TCP constituent phases (Figure 5b,c). The *c*-parameter was much more sensitive to the cation substitution in the case of the *β*-TCP counterpart. The unit cell expansion is explained by the partial substitution of Ca^2+^ ions with larger Sr^2+^ ions. 

In the case of Sr and Mg double doping, the lattice constants decreased compared to the BCP-6Sr material, as a result of partial substitution of Ca^2+^ with the smaller Mg^2+^ ions. This reduction was rather small for HA and quite significant for *β*-TCP, for which the lattice constants reached values lower than those recorded for the undoped BCP; wherefrom was suggested that Mg entered preferentially into the *β*-TCP lattice. Based on the crystallite size growth of *β*-TCP in the presence of Mg and the decrease of microstrain (local distortions) in *β*-TCP (data not presented), it can be concluded that Mg relaxes the *β*-TCP lattice. Therefore, the variation of the lattice parameters indicated that Sr and Mg entered the HA and *β*-TCP lattices on Ca positions, demonstrating that the described manufacturing process led to successful doping in the grains’ volume and not only to surface adsorption. Thereby, the role of HA destabilizer of Mg (being known to reduce the temperature of the HA → *β*-TCP transition [83,84]) has been anticipated based on scientific literature and confirmed within this study by the increase of *β*-TCP content (and decrease of HA) (Figure 4b) when adding Mg, along with the increase of crystallite size (Figure 5a) and the reduction of microstrain in *β*-TCP. Previous reports link this to the smaller ionic radius of Mg^2+^ (0.65 Å) with respect to Ca^2+^ (0.99 Å) having a consequence contraction of HA lattice parameters, which gives rise to lattice strain in HA, favouring its decomposition [83,84]. Furthermore, Mg was shown to be an efficient additive for the stability of *β*-TCP and the increase of the high-temperature limit of the *β*-TCP phase [57,85], possibly opening routes for the better densification of BCPs at superior sintering temperatures. Such experimental evidence could prove useful for the future large-scale fabrication of BCP ceramics with designed HA/*β*-TCP phase fraction. To the best of our knowledge, no results concerning the structure of bi-phasic calcium phosphate (HA + *β*-TCP) double-doped with Sr and Mg have been reported to date. 

**Figure 6:**
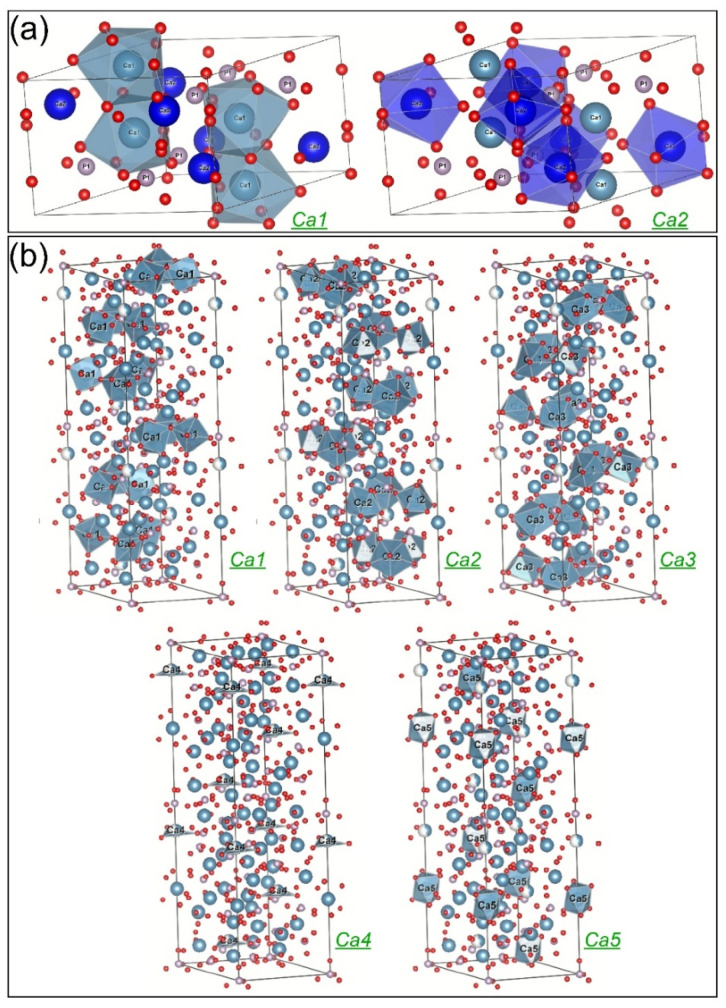
The unit cell of (**a**) HA (hexagonal, space group P63/m, a = b = 9.43 Å, c = 6.88 Å), emphasizing the two types of Ca sites displayed with their coordination polyhedra, and (**b**) *β*-TCP (rhombohedral, space group R3cH, a = b = 10.43 Å, c = 37.40 Å) highlighting the five types of Ca sites displayed with their coordination polyhedra. Note: The graphical representations were generated using the VESTA software (https://jp-minerals.org/vesta/en/download.html (accessed on 12 July 2022)).

Another problem to be elucidated is the proportion (probability) in which these cation substitutions occur. The distribution of the doping elements on various crystallographic positions could influence the solubility and other properties of the doped materials. The Rietveld analysis allows the estimation of the probability of finding one or another atom in a given crystallographic site, expressed by the so-called occupation factors of the sites. There are two nonequivalent Ca positions in the HA lattice, Ca1(4) and Ca2(6) (Figure 6a), while in the case of *β*-TCP there are five such positions, Ca1(18), Ca2(18), Ca3(18), Ca4(6), and Ca5(6) (the site multiplicities are given in the parentheses) (Figure 6b). One can also note that in the equilibrium structure of pure *β*-TCP the Ca4 crystallographic site is only partially (43%) occupied. 

The procedure for estimating the probability of Sr and Mg substitution for Ca atoms from different crystallographic sites is presented in the following. In the case of the single Sr substitution (sample BCP-6Sr), Sr was added alongside Ca to all (nonequivalent crystallographic) Ca sites of both HA and *β*-TCP. The structure refinement started from two structures from the Inorganic Crystal Structure Database (ICSD): HA according to the ICSD card no. 203027 [86], and *β*-TCP according to the ICSD card no. 97500 [87]. All site positions (*x*, *y*, *z*) were kept fixed at the values given in the ICSD references (trying to refine them did not improve the fit). The temperature factors were fitted to an overall value, the same for each element and site. To refine the occupation factors for Ca and Sr, *occCa* and *occSr*, we imposed to each Ca site the constraint *occCa* + *occSr* = 1 (that is, the sites are occupied either by Ca or Sr, and vacancies are excluded), and *occSr* were refined. This has been applied for all Ca sites except for Ca4 in *β*-TCP, where *occCa* + *occSr* = 0.43, due to the implicit vacancies in the pure *β*-TCP. The occupation factors obtained for the single Sr doping (sample BCP-6Sr) were taken as initial values for refining the co-substitution with Sr and Mg (sample BCP-6Sr2Mg). After adding Mg to each Ca site, we used the following algorithm: The sum *y = occCa + occSr*, was constrained to a subunitary value, and for the occupation factor of Mg, *occMg*, was imposed *1*—*y* as the upper limit. Then the procedure was repeated for *y* values, varying in steps. The best solution was the one that gave the best value for the fit quality parameter.

The Rietveld structure refinement converged toward the following solutions:

- In HA, Sr substituted Ca with a probability of about 10% on both positions. In comparison, Mg entered the Ca1 site replacing 10% of Ca from these positions and only 1% of Ca2. It is generally believed that larger-sized cations tend to have a higher coordination number with the surrounding anions, while smaller-sized cations prefer to occupy smaller coordination sites [88]. Indeed, Ressler et al. [87] showed experimentally that Sr can occupy preferentially Ca1 sites in HA, but our results have not confirmed this, since similar probabilities for Sr to occupy the nine-coordinated Ca1 and the seven-coordinated Ca2 sites in HA were found. However, if one considers only the first nearest neighbouring oxygen atoms, both positions of Ca in HA are basically six-coordinated [89], which may support our result on Sr site affinity in HA. The preference of the smaller Mg ion for Ca1 is in an even greater contradiction with the above-presented conventional expectations. However, Ren et al. [90] obtained by simulation that the substitution of Mg for Ca1 sites in HA is energetically favoured, while Matsunaga [88] predicted theoretically a preference of Mg for Ca2 position in HA. On the other hand, it is reasonable to accept that the substitution preferences of Mg are different when there is competition with Sr, as in our case.
- In *β*-TCP, Sr behaved differently, when used as a single dopant than when associated with Mg. As a single dopant, Sr preferentially replaced Ca4 (~70%) and Ca3 (25%), and ~13% of Ca1, Ca2, or Ca5. The preference of Sr for Ca3 and Ca4 sites in *β*-TCP agrees with the theoretical predictions of Matsunaga et al. [91]. The affinity of Sr for the Ca4 sites has been also experimentally demonstrated in the case of *β*-TCP in several studies [56,92,93]. In the presence of Mg, Sr apparently substituted all Ca4 atoms, possibly occupying also part of the vacant positions of this crystallographic site. Mg showed a strong preference for the Ca3 (~35%) and Ca1 (~25%) sites, while Sr substituted about 15% of Ca on Ca2 sites, and ca. 5% from the Ca1, Ca3, and Ca5 sites. Therefore, the preference of Sr for Ca4 sites in *β*-TCP was enhanced in the presence of Mg, (i.e., BCP-6Sr2Mg samples), while the share of Ca3 and Ca1 sites decreased as an [56,92,93] effect of competition between Sr and Mg cations.

Concluding, despite the mathematical difficulty of refining such a complex structure (bi-phasic, double substitution for Ca sites, seven nonequivalent Ca sites), we found agreements with site-occupation data obtained by other researchers on pure HA or *β*-TCP. One of the original results is that in the case of the double-doping with Sr and Mg, the substitution preferences are different compared to the single Sr doping.

Apart from the mathematical uncertainty, which may lead to multiple solutions, there could be other reasons for observed discrepancies between the *ab initio* and experimental results and, generally, between the published data concerning cationic Ca-site substitutions in the HA discussed in reference [89]. 

The Rietveld refinement gave huge substitution ratios for Ca in both the Sr and Mg cases which were unrealistic with respect to the designed (and EDXS verified) Sr and Mg concentration in the materials. However, these results (supposing that the distribution of dopants at different Ca sites is correctly estimated) can still be used to evaluate how Sr and Mg are distributed between the HA and *β*-TCP phases. This depends on the one hand on how many HA and *β*-TCP molecules are, how many Ca atoms they contain, and on the other hand on the affinity of dopants to substitute them.

**Figure 7:**
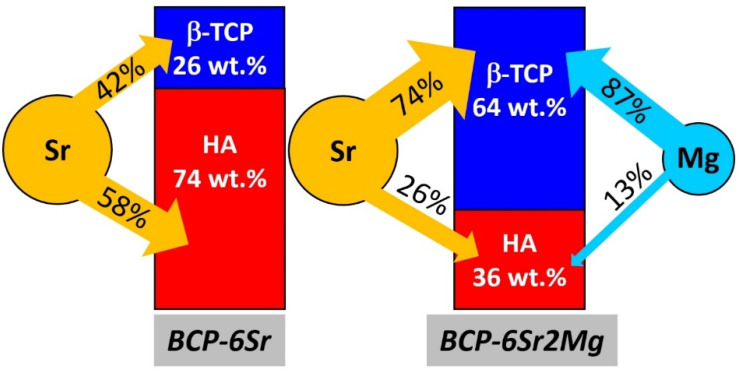
Cation occupation shares of Sr and Mg in the Ca sites of the HA and *β*-TCP constituents of the doped BCP ceramics.

For instance, in the case of BCP-6Sr, the Rietveld refinement yielded a share of ~74 wt.% and ~26 wt.% for the HA and *β*-TCP, respectively. As the wt.% of Ca in the undoped or pristine HA molecule (39.8 wt.%) is almost the same as that in the undoped *β*-TCP (38.7 wt.%), it results that in this material ~75% of total Ca pertains to HA, whilst the rest of ~25% to *β*-TCP. On the other hand, by calculating the number of Sr atoms which enter the HA unit cell by summing the respective substitution probabilities, and doing the same for *β*-TCP, after renormalization, one obtains that 58% of Sr entered HA and the rest of 42% was incorporated into *β*-TCP. Consequently, for BCP-6Sr, 58% of the total Sr atoms entered the HA lattice, which contains 75% of the total Ca atoms of the material, while the rest of 42% Sr entered *β*-TCP that contains the rest of 25% Ca atoms (see the diagram in Figure 7). By defining the affinity toward a given BCP phase as the ratio of the Sr atoms to the host Ca atoms, it is obtained that for the BCP-6Sr specimen the affinity of Sr for HA is 58/75 = 0.8, while for *β*-TCP it is 42/25 = 1.7.

Similarly, one can obtain that in the case of BCP-6Sr2Mg, 26% of total Sr and 13% of total Mg entered HA which contains 37% of total Ca atoms, while the rest of 74% Sr and 87% Mg was incorporated into *β*-TCP, which contains 63% of total Ca (Figure 7). Therefore, when there was a competition between Sr and Mg to substitute Ca in a mixture of HA and *β*-TCP, the affinity of Sr for HA was almost as in the case of BCP-6Sr, of 26/37 = 0.7, while the one for *β*-TCP was diminished to 74/63 = 1.2 (Figure 7). Mg had an affinity of 14/37 = 0.4 for HA, and of 87/63 = 1.4 for *β*-TCP (Figure 7). 

Concluding, both Sr and Mg have a much larger affinity toward Ca sites in *β*-TCP lattice than for Ca in HA. In BCP-6Sr2Mg, the competition between Sr and Mg is mostly for the Ca sites in *β*-TCP. To the best of our knowledge the incongruent cation doping in the BCP components HA and *β*-TCP has not been yet reported. Such findings could be of paramount importance for the future development of BCP-based bone graft substitutes as the control over the release of therapeutic cations incorporated into the two BCP constituents, with quite dissimilar dissolution rates, can foster paths toward both predictable *in vivo* performances of such medical devices and personalized medicine. 

### 3.2. Preliminary In Vitro Cytocompatibility Assessments

#### 3.2.1. Cell Proliferation

When working with a cell culture, such as hFOBs, that is not terminally differentiated, for long culturing times, some cells can undergo differentiation in contact with certain materials, leading to a heterogeneous cell population. Since differentiated and undifferentiated cells have different organelle numbers and capacities for differentiation, errors could arise when using a single type of proliferation assay. Thus, in order to gauge more accurately the cell proliferation induced by the BCP-based scaffolds, we decided to employ three different approaches: (i) a usual MTS assay, (ii) an LDH test, and (iii) an acridine orange (AO) approach. (i) In the MTS assay, the proliferation of cells is estimated indirectly by measuring the cell enzymatic capacity to transform MTS into the blue formazan product. Some conditions can interfere with the test and induce a difference between the real proliferation and the one calculated by the total metabolic capacity of producing formazan. For instance, the presence of specific metallic ions leached from the material could alter the cellular metabolic processes and induce errors in cell proliferation estimation. (ii) LDH, an intracellular enzyme, quite resilient to inactivation, being mostly used to determine cell death by investigating its quantity leaked from dead cells in the growing medium, was used as a backup verification for cell proliferation. (iii) Another venue of cell proliferation assessment was the use of acridine orange, a fluorescent chemical that enters cells and bind to nucleic acids such as DNA and RNA. The AO assay, in contrast with the two previously mentioned ones, which infer protein activity, measures another type of cellular class of substances, i.e., the nucleic acids. By collective assessment, the information provided by these three independent tests was believed to foster a more relevant and complete image of cell proliferation, with smaller room for errors.

##### MTS-Based Cell Proliferation Assay

**Figure 8:**
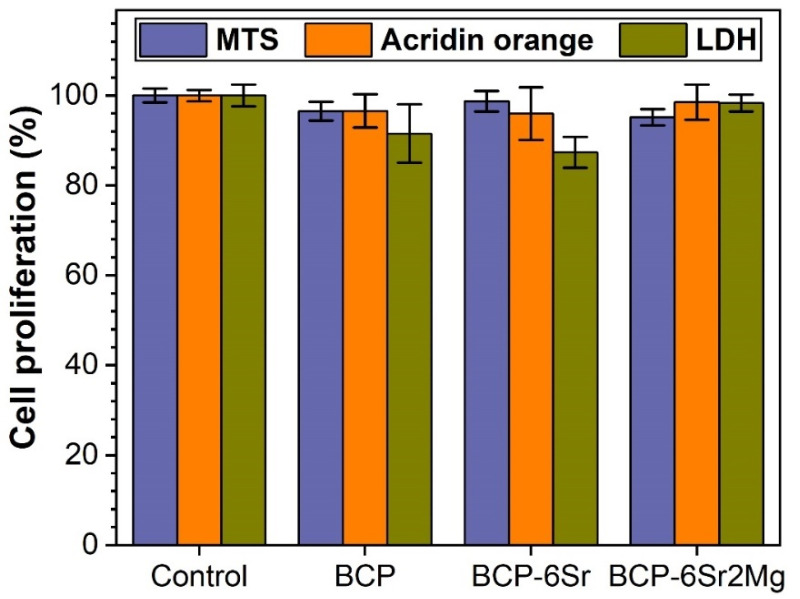
Cell proliferation of hFOB 1.19 grown on the BCP-based scaffolds, as assessed by MTS, LDH and Acridine orange assays after 14 days of culturing.

The MTS tests indicated slightly smaller cell proliferation values for the BCP-based scaffolds with respect to the cell adhesive polycarbonate control surface (see Figure 8). This constituted a first indication of the good cytocompatibility of the robocasting-printed scaffolds. The marginally smaller values obtained on scaffolds (i.e., 95.1–98.7% vs. 100% for adherent cell culture dedicated polycarbonate, *p* < 0.05) could arise from the adsorption of small quantities of formazan product on the surface of the BCP-based ceramics.

##### LDH-Based Cell Proliferation Assay

The LDH assay is often used to quantify the presence of dead cells in a given cell compatibility experiment. However, this specific intracellular enzyme can be also used to assess, with good results, the proliferation of a cell population. A disadvantage of this assay is that it requires the lysis of the entire cell population in order to release in the medium the enzyme to be later quantified. The quantity of the LDH is proportional to the cell number, thus giving a measure of cell proliferation. The data provided by the LDH assay showed almost similar results for polycarbonate control and all types of scaffolds included in this study. The smallest LDH cell proliferation values were recorded for BCP-6Sr scaffolds (i.e., 87.3%), whilst the highest for the BCP-6Sr2Mg (i.e., 98.3%), furthermore, they were situated very close (with no significant statistical differences, *p* > 0.05) to the cell adherent biological control (Figure 8). Although the spread of datapoints is larger for this technique with respect to the MTS assay, no statistically significant differences (*p* > 0.05) were recorded between the undoped and cation-doped BCP scaffolds, to eloquently sustain a smaller cytocompatibility for a specific situation. 

##### AO-Based Cell Proliferation Assay

In order to better gauge the cell proliferation in this difficult environment, the authors decided to employ an easier method to assess the total quantity of RNA in the cells by using a fluorescent tracer that permeates in live cells and couples with cytoplasmic messenger RNA. Upon RNA coupling it drastically changes its fluorescence parameters, i.e., excitation maximum at 460–480 nm and emission maximum at 535 nm. All studied scaffolds elicited cell proliferation results slightly lower compared to the biological control. The smallest values were registered for the BCP-6Sr scaffolds (i.e., 95.9%), and the highest for the BCP-6Sr2Mg (i.e., 98.5%) (Figure 8). These small differences were not statistically significant (*p* > 0.05) with respect to the biological control. This demonstrated a good interaction between the hFOB 1.19 cells and the BCP-based scaffolds, highly similar to the one encountered for adherent cell culture dedicated polycarbonate surface.

##### Integrative Analysis of the Cell Proliferation Assays

As previously debated, in order to attain a full proof image of the cell proliferation in scaffolds made of biomaterials leaching specific ions that could inhibit enzymes or adsorb certain substances or reaction products on their surface, a multiple test-approach should be employed. The combined analysis of the three different cell proliferation assays demonstrated that the proposed innovative scaffold concepts sustain a good *in vitro* proliferation of human osteoblast cells, comparable with that induced by the standard cell culture dedicated surfaces. Some differences were seen across the three types of performed cell proliferation assays (i.e., MTS, LDH, and AO) for a given type of structure. The LDH approach was the one with a greater datapoint spread and a larger standard deviation. All presented techniques complement each other, are easy to perform in an entry-level biofacility, are cost-effective, and together generate a good quantification of cell proliferation even on challenging biomaterial scaffolds. Therefore, this study could serve as a road map for future similar endeavours and it aimed to identify an easy, low-error experimental path to quantify cell proliferation and cell compatibility of porous scaffolds.

#### 3.2.2. Cell Death

**Figure 9:**
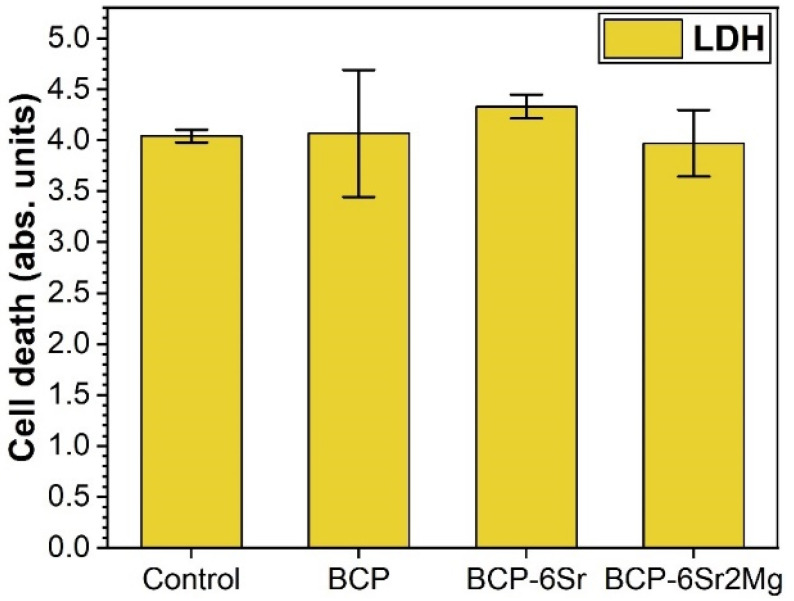
Cytotoxicity of the BCP-based scaffolds assessed by an LDH test after 14 days of hFOB 1.19 cell culturing.

In order to fully understand the behaviour of the hFOB cells, in parallel with the proliferation assays, cell death was investigated. To infer the fraction of dead cells, LDH (an intracellular enzyme that leaks in the cell culture medium upon cell death) was quantified in the cell culture medium, and the result was compared to the total LDH quantity present in the cells after 14 days in culture. This assay showed an average cell death between 3.97% and 4.33% for the cells grown on the scaffolds (lower for the BCP-6Sr2Mg ones), thus similar to the one determined by the biological control (4.04%) (Figure 9). However, the differences were statistically insignificant (*p* > 0.05, one-way ANOVA followed by Tukey’s post hoc test). Identical results were also yielded using the Student’s *t*-test, with one sole exception, control vs. BCP-6Sr situation (*p* = 0.033). This intermethod dissimilarity might be determined by the lower number of replicates (3) employed for the cell death experiments. Thus, the data indicated good cytocompatibility and a small intervention of the scaffolds on cell mortality.

Overall, the best cytocompatibility was observed for the double-doped BCP-6Sr2Mg scaffolds (also presenting the highest *β*-TCP content (i.e., ~64 wt.%)), suggesting the positive role of the concomitant leaching of the Sr and Mg ions.

#### 3.2.3. Cell Morphology

**Figure 10:**
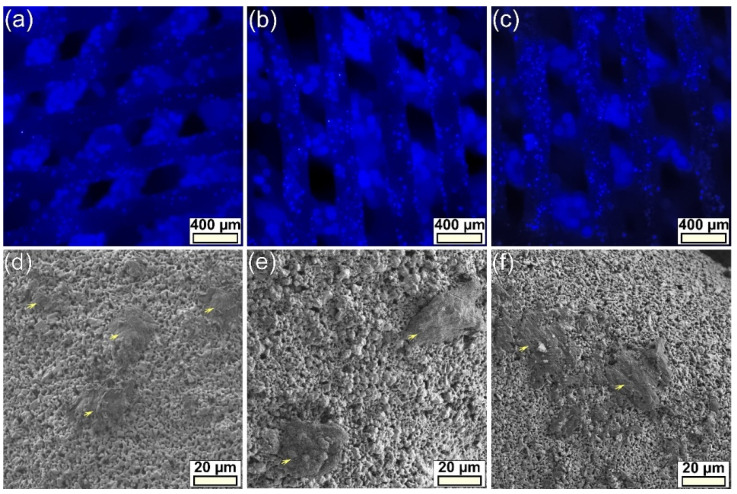
hFOB 1.19 cell morphology evidenced by (**a**–**c**) epifluorescence (cell nuclei are blue-stained with DAPI) and (**d**–**f**) electron microscopy of the (**a**,**d**) BCP-, (**b**,**e**) BCP-6Sr, and (**c**,**f**) BCP-6Sr2Mg-based scaffolds after 14 days of culturing. Cells were indicated on the micrographs with yellow arrows.

The BCP-based scaffolds were found to exhibit a high autofluorescence for the FITC and PE filter cubes, and a lower autofluorescence one for the one with DAPI. As such, only the cell nuclei counterstained with DAPI could be properly assessed. Cell nuclei, observed for the first three layers of scaffolds, presented an even distribution inside the macroporous constructs (Figure 10a–c). The cells seemed to be present on the entire specimen surface, although it was hard to pinpoint with the appropriate filter, the actin cytoskeleton stained with Phalloidin AlexaFluor546 has proven to be elusive to photography, due to the high autofluorescence of the scaffolds (leading to a very poor signal-to-noise ratio).

Further, the scaffold samples, prepared as described in Section 2.4.8, were investigated by FE-SEM on both top surfaces, as well as in cross-section on fractured (along their height) scaffolds to visualize cells on more deposited layers. FE-SEM analyses indicated a good 3D spread of cells inside the scaffold and a good attachment of the cells to the constituting ceramic filaments (Figure 10d–f), in agreement with the positive quantitative cytocompatibility results (Figure 8 and Figure 9).

Obtaining cytocompatibility responses (confirmed by four independent assays) analogous to those of the standard cell culture control can be considered an excellent result. This should be furthermore stressed since the cytocompatibility response of the standard adherent cell treated surface was matched by the proposed 3D constructs with complex spatial architecture inducing more challenging adherence conditions for cells. These encouraging cytocompatibility results are prerequisites for future studies targeting the impact of cation-substituted BCP-based scaffolds on stem cells, with emphasis on inter-relations between stem cell and their niche, cell proliferation, differentiation, and preservation of a stem cell pool.

## 4. Conclusions

Undoped and Mg- and/or Sr-substituted bi-phasic calcium phosphate (HA + *β*-TCP) macroporous scaffolds with high solid loading (of ~51–54 vol.%) were successfully fabricated with robocasting, a simple, yet promising 3D printing technique, employing hydrothermally synthesized BCPs source materials with a median particle size of ~1.2–1.4 μm and a low organic additives consumption. Single (Sr 6 at.%) and double (Sr 6 at.% and Mg 2 at.%) nominal cation substitutions in BCP were explored. Only in the case of Sr, a ~13% relative concentration decrease was experimentally emphasized, suggesting that the solubility limit of Sr was reached for the selected bi-phasic bioceramic system.

The structural effect of the single (Sr) and double (Sr and Mg) doping on the phase—HA and *β*-TCP—constitution was explored using FTIR-ATR spectroscopy and XRD and aimed to gain relevant technical information which might pave the road toward BCPs with improved and highly-predictable biological performance. Mg and Sr were found to substitute Ca predominantly in the *β*-TCP (at shares of 74% and 87%, respectively), with Mg playing a destabilizer role on HA, and Mg and Sr playing stabilizer roles on *β*-TCP, leading to the highest *β*-TCP concentration (~64 wt.%) in the double doped BCP. 

Integrating the information obtained by the (MTS, LDH, and AO) cell proliferation and (LDH) cell death assays with the epifluorescence and electron microscopy experiments one can conclude that all BCP-based scaffolds were highly cytocompatible with respect to human osteoblasts, with emphasis on the Sr and Mg double doped BCP ones, which remarkably elicited *in vitro* biological performances close to one of the standard surfaces dedicated for cell cultures. Thus, possibilities emerged that such robocasting-printed macroporous scaffolds could serve as viable future solutions for osseous tissue reconstruction and substitution. However, further insightful biological testing, employing cell differentiation experiments coupled with ion release investigations are needed and are envisaged to be performed in the future, to complete the picture of their biofunctional potential. 

## Figures and Tables

**Table 1:** Expected composition and coding for the prepared biphasic calcium phosphate samples.

Sample Code	Sr (at.%)	Mg (at.%)
BCP	0	0
BCP–6Sr	6	0
BCP–6Sr2Mg	6	2

**Table 2:** Formulations of the bioceramic pastes used for the 3D printing of the macroporous scaffolds using the robocasting method.

Sample Code	Initial Solid Loading (vol.%)	Mass Concentration (in wt.%) of Additives *			Final Solid Loading (vol. %)
		Dispersant (35 wt.% aq. Sol.)	Binder (33 wt.% aq. Sol.)	Coagulant (10 wt.% aq. Sol.)	
BCP	~53.9	0.57	2.00	0.04	~50.8
BCP—6Sr	~54.8	0.40	2.00	0.07	~51.4
BCP—6Sr2Mg	~57.8	0.38	2.00	0.05	~54.2

* Relative to the loaded bioceramic, considering a theoretical density of the BCP of 3.15 g/cm^3^.

## Data Availability

Not applicable.
